# Santamarine Shows Anti-Photoaging Properties via Inhibition of MAPK/AP-1 and Stimulation of TGF-β/Smad Signaling in UVA-Irradiated HDFs

**DOI:** 10.3390/molecules26123585

**Published:** 2021-06-11

**Authors:** Jung Hwan Oh, Junse Kim, Fatih Karadeniz, Hye Ran Kim, So Young Park, Youngwan Seo, Chang-Suk Kong

**Affiliations:** 1 Marine Biotechnology Center for Pharmaceuticals and Foods, College of Medical and Life Sciences, Silla University, Busan 46958, Korea; wjdghks0171@naver.com (J.H.O.); karadenizf@outlook.com (F.K.); cskong@silla.ac.kr (C.-S.K.); 2 Division of Marine Bioscience, Korea Maritime and Ocean University, Busan 49112, Korea; wnstp93@naver.com; 3 Department of Food and Nutrition, College of Medical and Life Sciences, Silla University, Busan 46958, Korea; gf5335@naver.com (H.R.K.); qkrthdud0409@naver.com (S.Y.P.)

**Keywords:** human dermal fibroblast, MAPK, photoaging, reynosin, santamarine, TGF-β, UVA

## Abstract

Chronic UVA exposure results in elevated reactive oxygen species in skin which leads to photoaging characterized as upregulated matrix metalloproteinase (MMP)-1 and loss of collagen. Therefore, natural antioxidants are hailed as promising agents to be utilized against photoaging. In the current study, reynosin and santamarine, two known sesquiterpene lactones isolated from *Artemisia scoparia*, were analyzed for their anti-photoaging properties in UVA-irradiated human dermal fibroblasts (HDFs). Results showed that UVA irradiation (8 J/cm^2^) upregulated the MMP-1 secretion and expression, and suppressed collagen production, which were significantly reverted by santamarine treatment (10 µM). Although both reynosin and santamarine exhibited ROS scavenging abilities, reynosin failed to significantly diminish UVA-stimulated MMP-1 release. UVA-irradiated HDFs showed increased collagen production when treated with santamarine. As a mechanism to suppress MMP-1, santamarine significantly suppressed the UVA-induced phosphorylation of p38 and JNK and nuclear translocation of p-c-Fos and p-c-Jun. Santamarine promoted collagen I production via relieving the UVA-induced suppression on TGF-β and its downstream activator Smad2/3 complex. Antioxidant properties of santamarine were also shown to arise from stimulating Nrf2-dependent expression of antioxidant enzymes SOD-1 and HO-1 in UVA-irradiated HDFs. In conclusion, santamarine was found to be a promising natural antioxidant with anti-photoaging properties against UVA-induced damages in HDFs.

## 1. Introduction

The structure, function and appearance of human skin are being threatened constantly by several hazardous environmental factors. Ultraviolet (UV) irradiation is one of those factors that has notable detrimental effects on skin including cancer, sunburn, inflammation and photoaging [1]. Studies have suggested various pathways that UV irradiation implements its harmful effects on skin structure and function. Ultraviolet A (UVA) is one type of radiation that is emitted by sun together with UVB and UVC. Unlike UVB, UVA deeply penetrates the dermis skin layer where the connective tissues and blood vessels are located [2]. Therefore, UVA is a major cause of cell apoptosis and photoaging in the dermal fibroblasts. UVA irradiation was shown to notably increase the production of reactive oxygen species (ROS) in the dermis layer after a short time following the exposure [3,4]. Accumulation of ROS leads to progression of inflammatory response, apoptosis, and the speeding of aging-related damages in dermal fibroblasts [5]. Premature aging of the skin cells, also known as photoaging, is considered to progress via damaged extracellular matrix (ECM) over time [6]. Oxidative stress mediated by UVA radiation has been suggested to play a vital role in the induction of matrix metalloproteinase-1 (MMP-1) produced by the skin cells, including keratinocytes as well as fibroblasts [7]. UVA-induced elevated generation of ROS stimulates the expression and enzymatic activity of MMP-1, -3 and -9, which in turn causes the collagen to be rapidly digested [8,9]. Downregulated collagen production mechanism coupled with this rapid collagen degradation is crucially involved in the deformation of ECM, the main reason for formation of wrinkles, and other characteristics of prematurely aged skin [10]. In this context, many of the agents that induce skin cell apoptosis are oxidants or stimulators of cellular oxidative metabolism, whereas many inhibitors or preventive agents against UVA-induced skin damage have antioxidant properties. 

**Figure 1:**
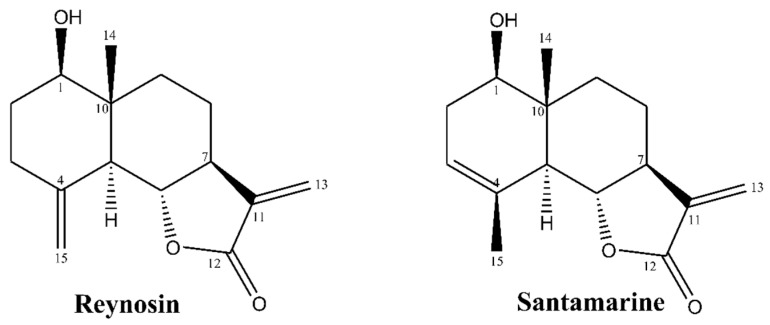
The chemical structures of reynosin and santamarine.

Nuclear factor E2-related factor 2 (Nrf2), the redox-sensitive transcription factor, is a master regulator of the cellular antioxidant defense against environmental damage, including UV exposure [11]. Nrf2 has been reported to play a beneficial role in protecting skin cells, including keratinocytes, fibroblasts, and melanocytes, against UV-induced oxidative damage and cellular dysfunction [12]. Thus, investigation of compounds targeting Nrf2-regulated antioxidant defense to combat oxidative stress and targeting collagen formation mechanism to replenish digested collagen deposits could provide insight into development of a promising pharmacological approach to help delay skin photoaging. Over the years, several antioxidants have been reported to be able to protect skin cells against UV-implemented harmful changes [13,14,15]. *Artemisia scoparia* is a widespread plant that belongs to a very large flowering plant family of *Asteraceae* and growing natively across Eurasia. Leaves and flowers of *A. scoparia* are referred in traditional medicine sources with activities such as diuretic, antiphlogistic and for treatment of hepatitis [16]. In addition, several studies reported antioxidant, insecticidal, phytotoxic and anti-inflammatory properties of *A. scoparia* as well as chemical constituents derived from it such as essential oils, flavonoids and coumarins [16,17,18]. Besides, the family *Asteraceae* which contains *A. scoparia*, includes many species where promising antioxidant molecules, especially sesquiterpene lactones were isolated [19]. In addition to antioxidant properties, these sesquiterpene lactones showed antimicrobial, anti-inflammatory, and anti-malarial activities in vitro [20]. In this study, the antioxidant potential and anti-photoaging mechanisms of reynosin and santamarine (Figure 1), two sesquiterpene lactones isolated from *A. scoparia*, were analyzed in UVA-irradiated human dermal fibroblasts (HDFs) in vitro.

## 2. Materials and Methods

### 2.1. Chemicals and Reagents

The chemicals used for plant extraction and compound isolation, and the companies from where they were obtained are as follow: Methylene chloride (D1602) from Samchun Chemicals (Seoul, Korea); *n*-butanol (6313050380) and methanol (73125) from Junsei Chemical (Tokyo, Japan); *n*-hexane (9304) from J. T. Baker (Phillipsburg, NJ, USA). Remaining reagents and their vendors for the experiments are as follow: Dimethyl sulfoxide (F041913), 3-(4,5-dimethylthiazol-2-yl)-2,5-diphenyltetrazolium bromide (F200220) and retinoic acid (F180103) from Cellconic (Seoul, Korea); radioimmunoprecipitation assay buffer (R0278) and 2′,7′-dichlorofluorescin diacetate (D6683) from Sigma-Aldrich Korea (Seoul, Korea).

### 2.2. Isolation and Characterization of Reynosin and Santamarine

**Scheme 1:**
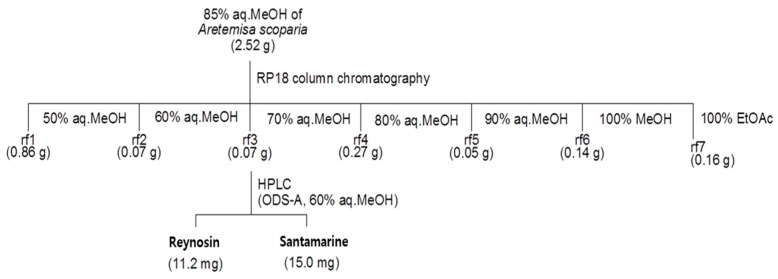
Isolation scheme of reynosin and santamarine from *A. scoparia* crude extract 85% aq. MeOH solvent fraction.

The two known sesquiterpene lactones, reynosin and santamarine, were isolated from *A. scoparia* crude extract. Briefly, the sample (100 g) of *A. scoparia* was air dried and ground. Ground plant material was extracted subsequently with 1 L methylene chloride (CH_2_Cl_2_) and 3 L methanol (MeOH) separately for 24 h at room temperature. Extracts from two solvents were concentrated in vacuo with a rotary evaporator, and crude extracts were obtained and combined. The combined crude extract from CH_2_Cl_2_ and MeOH extraction were partitioned between methylene chloride and water (H_2_O). Crude extracts were dissolved in a 1 L mix containing 1:1 (*v*/*v*) CH_2_Cl_2_ and H_2_O in a separating funnel. The mix was shaken vigorously for 5 min and the funnel was kept in room temperature for 4 h afterwards. This procedure was repeated two times until the separation was concluded. The methylene chloride layer was transferred to another flask and dried under reduced pressure using a rotary evaporator. The residue was dissolved and partitioned between 1 L mix (1:1, *v*/*v*) of *n*-Hexane and 85% aq. MeOH with same procedure above. Reynosin and santamarine were isolated from the 85% aq. MeOH fraction as shown in the scheme below (Scheme 1). Elucidation of the isolated compounds were carried out by comparison of spectroscopical data with published literature [21]. NMR spectral data were recorded on a Bruker Avance II NMR 900 spectrometer (Billerica, MA, USA) and obtained at the Korean Basic Science Institute (Taejeon, Korea).

### 2.3. Cell Culture and Viability Assay

HDF cells (C-12302; PromoCell, Heidelberg, Germany) were cultured in Fibroblast Growth Medium (C-23020, PromoCell) and kept in 37 °C incubators with a humidified (90% relative humidity) atmosphere containing 5% CO_2_ between the experiments.

Effects of UVA irradiation and samples on the viability of HDFs were investigated using common 3-(4,5-dimethylthiazol-2-yl)-2,5-diphenyltetrazolium bromide (MTT) assay procedures. Cells were seeded in 96-well plates (1 × 10^3^ cell/well) and incubated for 24 h, which was followed by the sample treatment or UVA irradiation. Viability of the cells was quantified after 24 h incubation. Briefly, wells were aspirated and supplied with 100 μL of MTT reagent (1 mg/mL). Plates were then incubated for 4 h. Formation of formazan salts was quantified by adding 100 μL dimethyl sulfoxide (DMSO) to each well, homogenizing and measuring the absorbance values at 540 nm with a GENios^®^ microplate reader (Tecan Austria GmbH, Grodig, Austria). Viable cell amount was then calculated as a relative percentage of the untreated or non-irradiated control well.

### 2.4. UVA Irradiation

HDFs were exposed to UVA (0, 2, 4, 6, 8 and 10 J/cm^2^) UVA via Bio-Sun biological UV–irradiation system (Vilber Lourmat, Marine, France) installed with 4× 30-Watt 365 nm UVA sources (T.40L, Vilber Lourmat). After UVA exposure was completed and the desired dose of UVA irradiation was achieved, HDFs were fed Fibroblast Growth Medium (C-23020, PromoCell) with/without samples for 24 h for the required assay.

### 2.5. DCFH-DA Cellular ROS Assay

The cellular generation of ROS was determined using an oxidizing radical species-sensitive dye: 2′,7′-dichlorofluorescin diacetate (DCFH-DA). HDFs were cultured in fluorescence microtiter 96-well plates and incubated for 24 h. Next, cell culture medium was loaded with 20 µM DCFH-DA in PBS and incubated for 20 min in the dark at room temperature. Cells were then treated with different concentrations of reynosin and santamarine and the plates were incubated for 1 h. After washing the cells with PBS three times, 500 µM H_2_O_2_ dissolved in PBS was added to the wells. The fluorescence intensity of the wells was read every 30 min for 3 h at an excitation wavelength of 485 nm and emission wavelength of 528 nm using a GENios^®^ microplate reader (Tecan Austria GmbH) to detect the 2′,7′-dichlorofluorescein (DCF) which was formed via oxidation of DCFH in the cells by ROS. Dose-dependent changes in DCF fluorescence intensity were plotted. Retinoic acid was used as a positive control.

### 2.6. Enzyme-Linked Immunosorbent Assay (ELISA)

UVA-induced changes in the MMP-1 secretion from irradiated HDFs were investigated by ELISA. HDFs were treated with reynosin and santamarine after UVA irradiation, and the contents of MMP-1 in the cell culture media was assessed following 24 h incubation using a commercial ELISA kit (Human Total MMP-1 DuoSet ELISA, cat no. DY901B; Human Pro-Collagen I alpha 1 DuoSet ELISA, cat no. DY6220; R&D Systems, Inc., Minneapolis, MN, USA) according to the manufacturer’s protocol. Retinoic acid was used as a positive control.

### 2.7. Reverse Transcription-Quantitative Polymerase Chain Reaction (RT-qPCR) Analysis 

HDFs were grown to confluence prior to UVA irradiation. Immediately after UVA exposure, cells were treated with santamarine for 24 h at 37 °C. Total RNA was extracted using AccuPrep Universal RNA Extraction Kit (Bioneer Corp. Daejeon, Korea) and the cDNA synthesis from total RNA (2 μg) was carried out with Cell Script cDNA master Mix (Cellsafe, Gyeonggi-do, Korea) in a T100 thermocycler (Bio-Rad Laboratories, Inc., Hercules, CA, USA). The following temperature protocol was used for reverse transcription: 42 °C for 60 min and 72 °C for 5 min. Subsequently, real time PCR was performed in TP800 Thermal Cycler Dice™ Real Time System (Takara Bio, Ohtsu, Japan) using Luna^®^ Universal qPCR Mix (New England Biolabs, Inc.) according to manufacturer’s protocol. The following primers were used for amplification: MMP-1 forward, 5′-GGAGCCAGCTCCCTCTATTT-3′ and reverse, 5′-GGCTACATGGGAACAGCCTA-3′; type I pro-collagen forward, 5′-AGAAGGAAATGGCTGCAGAA-3′ and reverse, 5′-GCTCGGCTTCCAGTATTGAG-3′; Nrf2 forward, and reverse; HO-1 forward, and reverse; SOD-1 forward, and reverse; β-actin forward, 5′-CCACAGCTGAGAGGGAAATC-3′ and reverse, 5′-AAGGAAGGCTGGAAAAGAGC-3′. The following thermocycling conditions were used for PCR: 30 cycles of 95 °C for 45 s, 60 °C for 1 min and 72 °C for 45 s. Retinoic acid was used as a positive control.

### 2.8. Western Blot

Detection of protein levels was performed using standard Western blot protocols. Briefly, HDFs were cultured in 6-well plates prior to UVA exposure. Following UVA irradiation, the cells were treated with samples for 24 whereas control cells were added same volume of growth medium in which the samples were dissolved. After 24 h treatment, the cells were homogenized using ice-cold 1 mL radioimmunoprecipitation assay (RIPA) buffer. The lysates were then transferred into tubes and centrifuged (13,000× *g*) at 4 °C for 15 min. The supernatants were used for protein detection. The nuclear fraction was extracted by NE-PER^TM^ nuclear extraction kit (Catalog No. #78835; Thermo Fisher Scientific). The protein contents of the extracts were analyzed with a BCA protein assay kit (Thermo Fisher Scientific, Waltham, MA, USA). The total or nuclear protein lysates containing same amount of protein (20 μg) were separated by SDS-PAGE (4% stacking and 10% separating gels). Proteins on gels were transferred to polyvinylidene fluoride membrane (Amersham Bioscience., Westborough, MA, USA) for immunoblotting with standard wet transfer protocols. Blocking of membranes was carried out by keeping membranes in 5% skim milk (*v*/*v* in TBS-T buffer) for 4 h on a shaking incubator. Blocked membranes were hybridized at 4°C overnight with antibodies against MMP-1 (cat. no. sc-6837; Santa Cruz Biotechnology, Inc., Santa Cruz, CA, USA), MMP-3 (cat. no. sc-21732; Santa Cruz Biotechnology), MMP-9 (cat. no. sc-393859; Santa Cruz Biotechnology), type I procollagen (cat. no. sc-8782; Santa Cruz Biotechnology, Inc.), p38 (cat. no. #8690; Cell Signaling Technology, Inc., Danvers, MA, USA), phospho(p)-p38 (cat. no. #4511; Cell Signaling Technology, In.c), JNK (cat. no. LF-PA0047; Thermo Fisher Scientific, Inc.), p-JNK (cat. no. sc-293136; Santa Cruz Biotechnology, Inc.), ERK (cat. no. #4695; Cell Signaling Technology, Inc.), p-ERK (cat. no. #4370; Cell Signaling Technology, Inc.), c-Jun (cat. no. sc-74543; Santa Cruz Biotechnology, Inc.), p-c-Jun (cat. no. sc-822; Santa Cruz Biotechnology, Inc.), c-Fos (cat. no. sc-7202; Santa Cruz Biotechnology, Inc.), p-c-Fos (cat. no. #5348s; Cell Signaling Technology, Inc.), TGF-β (cat. no. #3711; Cell Signaling Technology), Smad2/3 (cat. no. sc-133098; Santa Cruz Biotechnology), p-Smad2/3 (cat. no. sc-11769; Santa Cruz Biotechnology), Smad7 (cat. no. sc-101152; Santa Cruz Biotechnology), Smad4 (cat no. sc-56479; Santa Cruz Biotechnology), β-actin (cat. no. sc-47778; Santa Cruz Biotechnology, Inc.), and lamin B1 (cat. no. sc-374015; Santa Cruz Biotechnology, Inc.) diluted as suggested by the manufacturer in 1X TBS-T buffer containing 5% bovine serum albumin (*m*/*v*). Next, the membrane was incubated for 2 h at room temperature with horseradish-peroxidase-conjugated secondary antibodies specific to organism of primary antibodies. Membranes were stained with ECL kit (Amersham Bioscience) according to manufacturer’s instructions and the protein bands were imaged with CAS-400SM Davinch-Chemi imager (Davinch-K, Seoul, Korea). Retinoic acid was used as a positive control.

### 2.9. Immunofluorescence Staining

Detection of collagen I levels in UVA-irradiated HDFs were observed by immunofluorescence staining. HDFs were cultured on glass coverslips and exposed to UVA. Following UVA irradiation, cells were treated with santamarine. After 24 h of incubation, HDFs were fixed on glass coverslips and stained at 4 °C overnight with anti-collagen I antibody (cat. no. ab34710; Abcam, Cambridge, UK) conjugated with Alexa Fluor 488 (A-11008; Invitrogen, Carlsbad, CA, USA), and ProLong Gold Antifade Reagent with 4′,6-diamidino-2-phenylindole (DAPI) (cat. no. #8961; Cell Signaling Technology) for the nuclei highlighting. Fixation and staining of the cells were carried out using Immunofluorescence Application Solutions Kit (cat. no. #12727; Cell Signaling Technology), according to manufacturer’s instructions.

### 2.10. Statistical Analysis

Numerical results were given as average of three independent experiments ± SD run in triplicates where applicable, unless otherwise noted. Groups in same data series were subjected to one-way analysis of variance (ANOVA) with post-hoc Duncan’s multiple range test for statistical analysis (SAS v9.1, SAS Institute, Cary, NC, USA) and differences were defined significant at *p* < 0.05 level.

## 3. Results

### 3.1. The Characterization of Reynosin and Santamarine

Reynosin was obtained as an amorphous white solid. The characterization of reynosin was carried out through the results of ^1^H and ^13^C NMR (Table 1), ^1^H-^1^H COSY (Figure S1) and gHSQC and gHMBC (Figure S2) spectrum analyses and their comparison with published data [21].

Santamarine was isolated as an amorphous white solid. The characterization of santamarine was carried out through the results of ^1^H and ^13^C NMR (Table 2), ^1^H-^1^H COSY (Figure S3) and gHSQC and gHMBC (Figure S4) spectrum analyses and their comparison with published data [21].

Reynosin and santamarine were separated as a single peak at retention time 12.3 min and 19.0 min by reversed-phase HPLC (YMC ODS-A, 2 mL/min, 60% aq. MeOH), respectively. Their purities were more than 95 % on the basis of integration analysis of ^1^H NMR spectra (Figures S5 and S6).

### 3.2. Effect of UVA Irradiation and Sample Treatment on the Viability of HDFs

**Figure 2:**
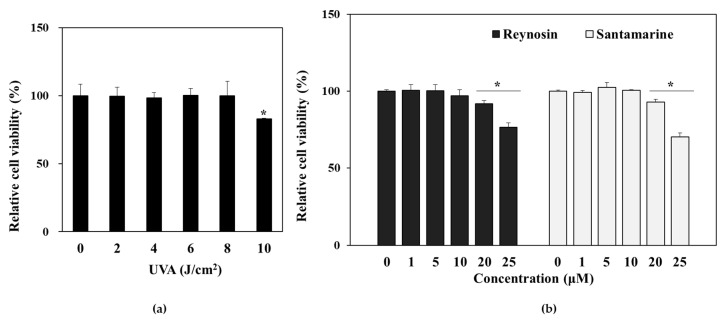
Effect of UVA (**a**) and reynosin and santamarine (**b**) on the viability of human derma fibroblasts (HDFs). Viability of the cells was measured after 24 h treatment with MTT assay. Viability was given as relative percentage of the untreated group. Values are means ± SD (*n* = 3). * *p* < 0.05, vs. untreated control.

The cultured HDFs were irradiated with ranging (0–10 J/cm^2^) doses of UVA and the cell viability was measured 24 h after the irradiation via MTT assay. Results showed that UVA exposure significantly decreased the viable cell amount starting from 10 J/cm^2^ (Figure 2a). Hence, the 8 J/cm^2^ dose was chosen for further assays as the highest UVA dose to induce UV-mediated intracellular change but not cell death in 24 h.

Next, HDFs were treated with varying concentrations (0–25 µM) of reynosin and santamarine in order to evaluate the cytotoxicity of the samples. Both samples decreased the cell viability significantly for the concentrations 20 µM and up (Figure 2b). Therefore, future assays were carried out using the concentrations 1, 5 and 10 µM of reynosin and santamarine as non-toxic doses. In addition, where applicable, retinoic acid was used as a positive control.

### 3.3. Measurement of Antioxidant Activity of Reynosin and Santamarine

**Figure 3:**
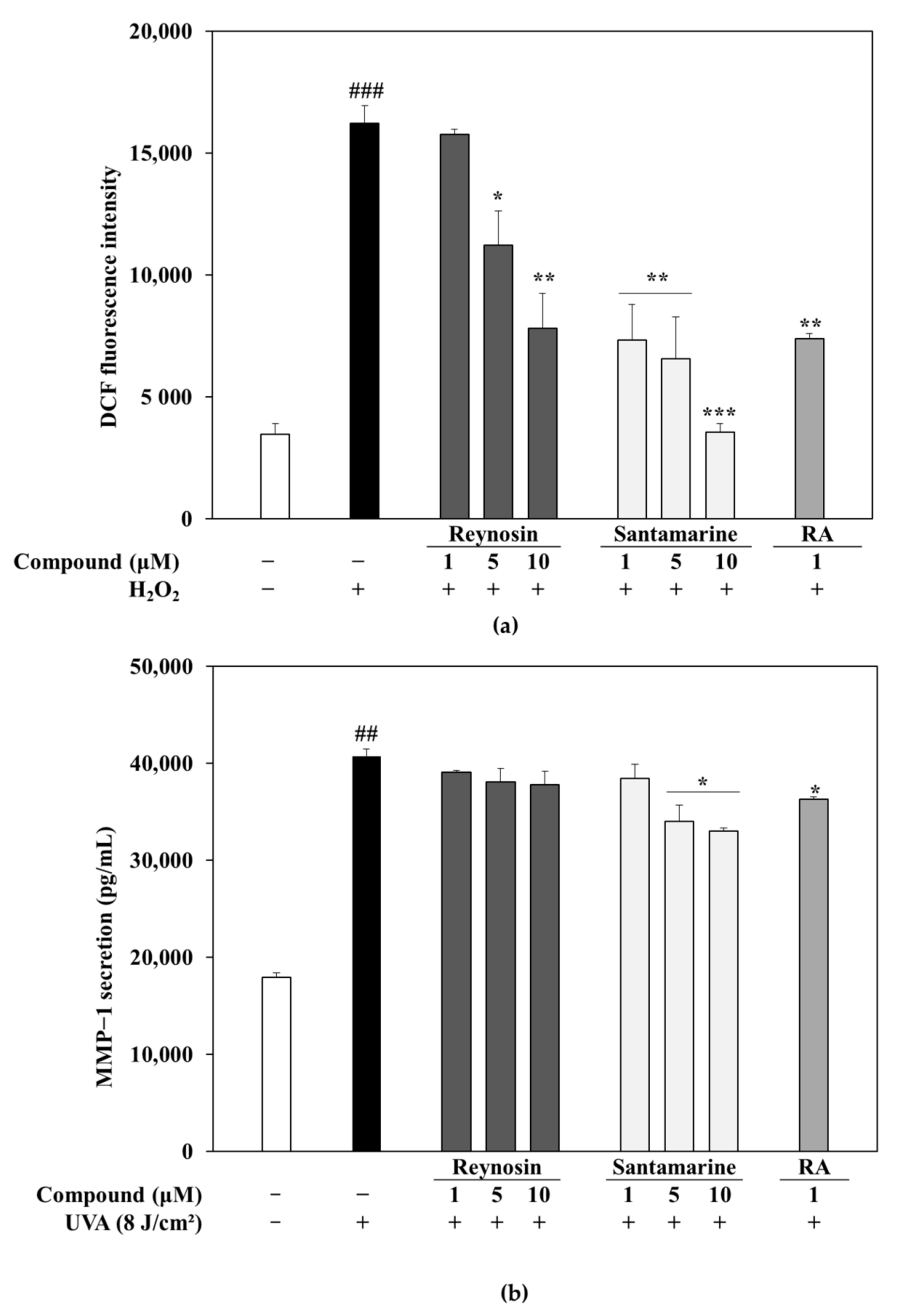
Effect of reynosin and santamarine on the generation of intracellular reactive oxygen species (ROS) and MMP-1 secretion in H_2_O_2_-treated and UVA-irradiated (8 J/cm^2^) HDFs, respectively. (**a**) ROS scavenging effects of reynosin and santamarine were analyzed after 24 h treatment by the DCFH-DA assay and given as the fluorescence intensity showing DCF generation via intracellular ROS. (**b**) MMP-1 secretion of the UVA-irradiated HDFs was measured by an ELISA kit following 24 h treatment. Values are means ± SD (*n* = 3). * *p* < 0.05, ** *p* < 0.01 and *** *p* < 0.001 vs. H_2_O_2_ or UVA only control. ^##^ *p* < 0.01 and ^###^ *p* < 0.001 vs. non-treated control. Retinoic acid (RA) was used as positive control.

The antioxidative capabilities of reynosin and santamarine were confirmed in HDFs using DCFH-DA fluorescent assay. As seen in Figure 3a, both reynosin and santamarine showed dose-dependent radical scavenging activity. Santamarine and reynosin decreased the ROS activity in HDFs with IC_50_ value of 4.04 and 11.28 µM, respectively.

### 3.4. Effect of Reynosin and Santamarine on UVA-Induced MMP-1 Secretion

UV-induced overexpression of MMPs is among the main reasons for skin to lose its elasticity and form wrinkles during photoaging progression. To assess the effects of reynosin and santamarine on the UVA-induced secretion of MMP-1 in cultured HDFs, the amount of MMP-1 enzyme in the culture supernatants was measured by ELISA kit. As seen in Figure 3b, santamarine decreased the MMP-1 secretion in UVA-irradiated HDFs in a dose-dependent manner. Although reynosin was able to suppress MMP-1 secretion to an extent, it was not as significant and notable as santamarine. At the concentration of 10 µM, santamarine treatment dropped MMP-1 secretion to 33.00 from 40.74 ng/mL of UVA irradiated only group. At the same conditions, reynosin treatment (10 µM) decreased MMP-1 levels to 37.76 ng/mL. Considering the effectiveness of the samples with both antioxidant and MMP-1 secretion inhibitory activities, santamarine was used for further mechanism elucidation assays due to potential it presents.

### 3.5. Santamarine Downregulated the UVA-Induced mRNA Expression of MMP-1, and Protein Expression of MMP-1, 3 and 9

**Figure 4:**
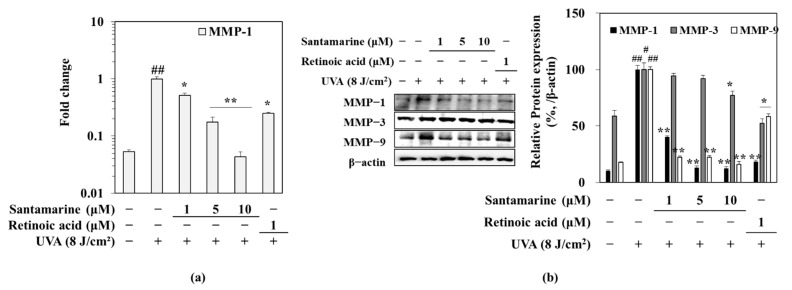
Effect of santamarine on the mRNA expression of MMP-1 (**a**) and the protein levels of MMP-1, MMP-3 and MMP-9 (**b**) analyzed by RT-qPCR and Western blot, respectively. UVA-irradiated (8 J/cm^2^) HDFs were treated with santamarine for 24 h and the harvested cells were used to measure the MMP levels. β-actin was used as a loading control. Values are means ± SD (*n* = 3). * *p* < 0.05 and ** *p* < 0.01 vs. UVA-irradiated untreated control. ^#^ *p* < 0.05 and ^##^ *p* < 0.01 vs. non-irradiated control. Retinoic acid was used positive control.

Following the evaluation of its effect on MMP-1 secretion, santamarine was tested for its ability to affect the expression of MMP-1 in mRNA level, assessed by RT-qPCR. As shown in Figure 4a, UVA exposure significantly increased the expression of MMP-1 in HDFs compared to non-irradiated group, whereas treatment with santamarine following UVA exposure markedly suppressed the MMP-1 mRNA expression in a dose-dependent manner. Similar trends were observed in protein levels. The UVA irradiation of HDFs resulted in elevated levels of MMP-1, -3 and -9 (Figure 4b). Presence of santamarine in culture medium following UVA irradiation decreased the MMP-1, -3 and -9 protein levels in HDFs in a dose-dependent manner.

### 3.6. Santamarine Suppressed UVA-Induced Activation of MAPK/AP-1

**Figure 5:**
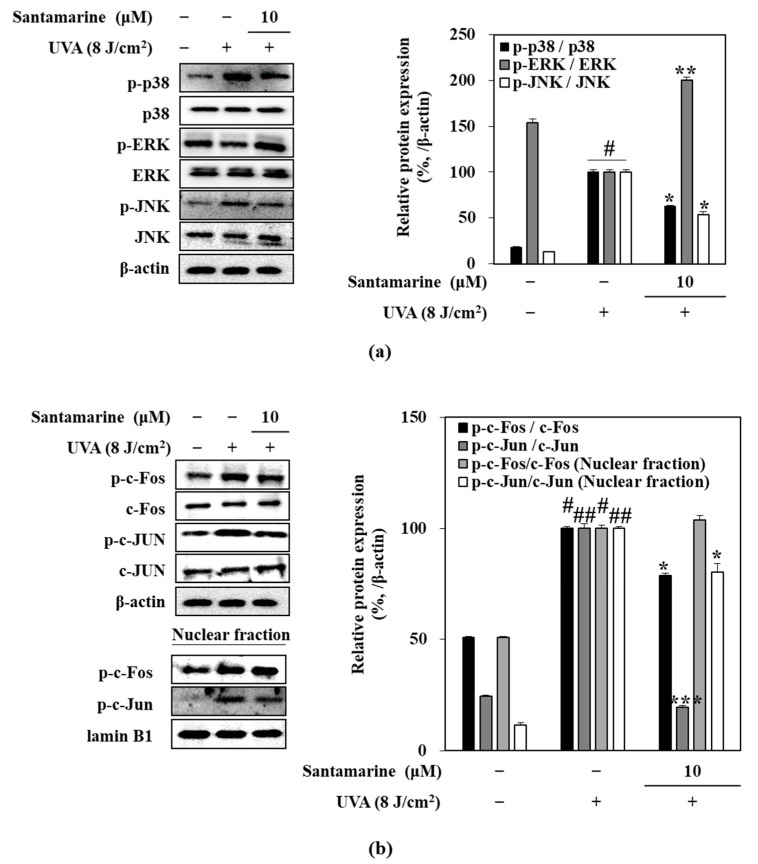
Effect of santamarine on the MAPK/AP-1 pathway activation. UVA-irradiated (8 J/cm^2^) HDFs were treated with santamarine for 24 h and the harvested cells were used to measure the total and phosphorylated levels of (**a**) p38, ERK and JNK MAPKs, and (**b**) c-Fos and c-Jun of AP-1 complex via Western blot. β-actin and lamin B1 were used as loading controls. * *p* < 0.05, ** *p* < 0.01 and *** *p* < 0.001 vs. UVA-irradiated untreated control. ^#^ *p* < 0.05 and ^##^ *p* < 0.01 vs. non-irradiated control.

The UVA-induced ROS generation activated MAPK signaling pathway which in turn facilitates the phosphorylation of c-Fos and c-Jun to form AP-1, a transcription factor responsible for MMP-1 expression. Figure 5a shows that UVA irradiation significantly increased the phosphorylation of p38 and JNK MAPKs, while it did not affect ERK activation. Treatment with 10 µM santamarine following UVA exposure resulted in suppression of p38 and JNK phosphorylation while ERK activation was further upregulated. To further confirm the effect of santamarine on MAPK/AP-1 pathway, the levels of c-Fos and c-Jun were also evaluated. The UVA-irradiated HDFs exhibited elevated levels of c-Fos and c-Jun activation (Figure 5b). In addition, translocation of AP-1 (formed by activated c-Fos and c-Jun) in nucleus was markedly stimulated by UVA exposure shown as increased p-c-Fos and p-c-Jun levels in nucleus (Figure 5b). Expectedly, santamarine (10 µM) significantly reduced the levels of p-c-Fos and p-c-Jun, as a suggested mechanism for its ability to suppress MMP production.

### 3.7. Santamarine Ameliorated UVA-Mediated Decrease in Type I Procollagen Expression

**Figure 6:**
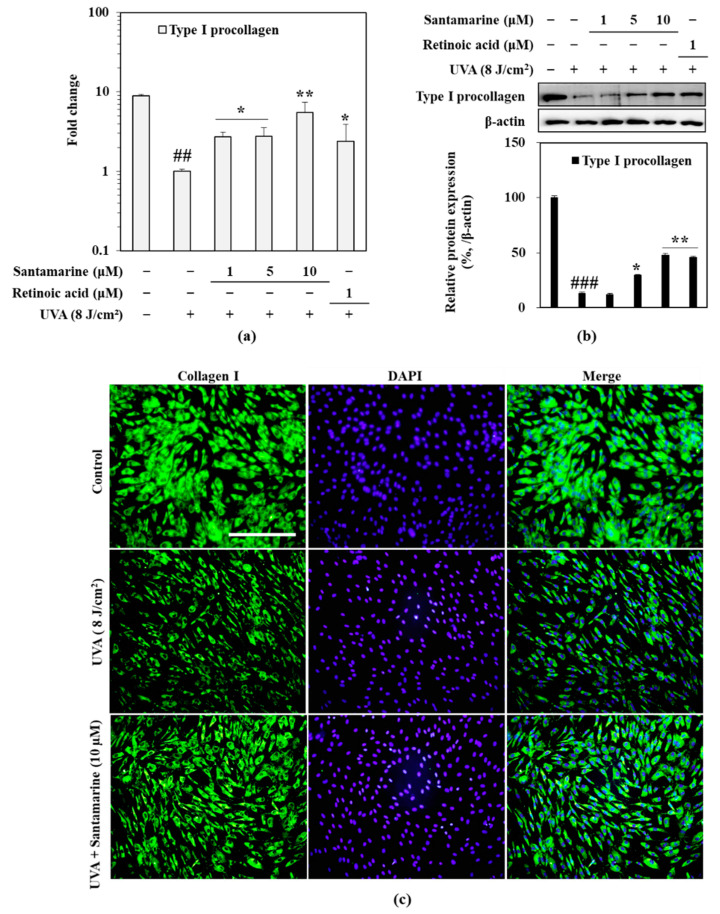
Effect of santamarine on the collagen production in UVA-irradiated (8 J/cm^2^) HDFs. Effect of santamarine on the mRNA expression (**a**) and protein level (**b**) of type I procollagen was analyzed after 24 h treatment post-UVA-irradiation via RT-qPCR and Western blot, respectively. β-actin was used as a loading control. Values are means ± SD (*n* = 3). (**c**) Fluorescence-stained images of the HDFs were taken after staining with FITC conjugated anti-collagen I antibody (green) and DAPI (blue) to highlight the nuclei. Scale bar, 100 μm. * *p* < 0.05 and ** *p* < 0.01 vs. UVA-irradiated untreated control. ^##^ *p* < 0.01 and ^###^ *p* < 0.001 vs. non-irradiated control. Retinoic acid was used as positive control.

Along with overexpression of MMPs, reduced collagen expression results in the deformed photoaged skin. To evaluate the effect of santamarine on collagen production in UVA-irradiated HDFs, mRNA and protein expression levels of type I procollagen were analyzed. HDFs irradiated with UVA showed downregulated mRNA (Figure 6a) and protein (Figure 6b) expression of type I collagen whereas HDFs treated with 10 µM santamarine exhibited alleviated type I procollagen expression in mRNA and protein level. 

The effect of santamarine on collagen levels in UVA-irradiated HDFs was further confirmed by fluorescent staining of cellular collagen. As shown in Figure 6c, UVA exposure significantly reduced the collagen amount in HDFs. Treatment with santamarine markedly relieved the UVA-induced decrease in collagen amount. 

### 3.8. Effect of Santamarine on TGF-β/Smad Pathway

**Figure 7:**
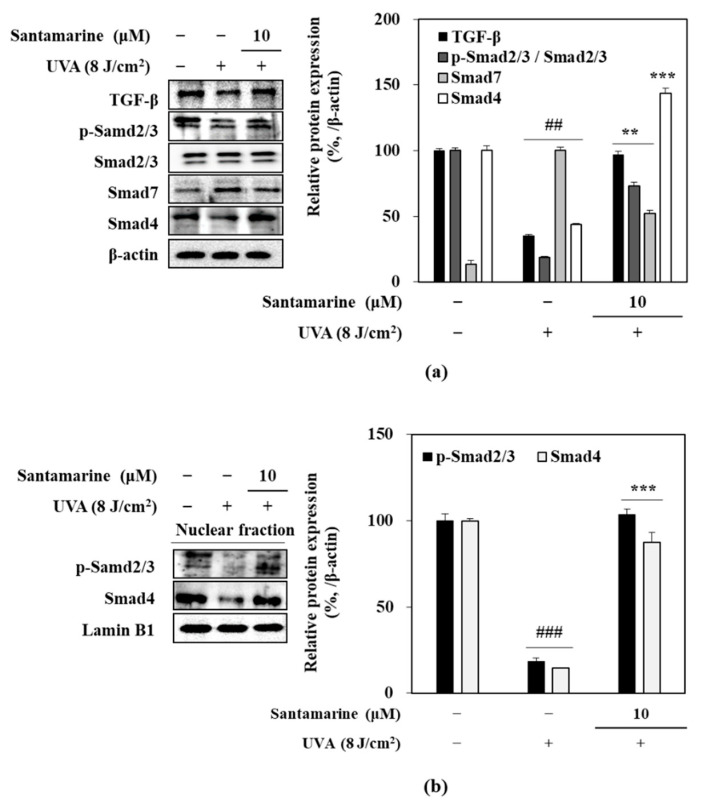
Effect of santamarine on TGF-β/Smad pathway activation. Total cell (**a**) and nuclear (**b**) levels of phosphorylated (p-) and total TGF-β/Smad signaling proteins were analyzed after 24 h treatment in UVA-irradiated (8 J/cm^2^) HDFs via Western blot. β-actin and lamin B1 were used as loading controls. ** *p* < 0.01 and *** *p* < 0.001 vs. UVA-irradiated untreated control. ^##^ *p* < 0.01 and ^###^ *p* < 0.001 vs. non-irradiated control.

To elucidate the type I procollagen production stimulatory effect of santamarine TGF-β and its downstream effectors Smad proteins were analyzed. As seen in Figure 7a, UVA irradiation suppressed the TGF-β protein, along with the activation of Smad2/3 complex. On the other hand, Smad7, inhibitor of Smad2/3 activation and nuclear translocation, was upregulated by UVA exposure. In contrast, santamarine treatment reversed the effects of UVA on TGF-β and Smad2/3 and Smad7. Santamarine presence alleviated the suppression of TGF-β levels and Smad2/3 phosphorylation while suppressing the Smad7 levels. The ameliorating effect of santamarine on UVA-induced suppression of TGF-B-mediated collagen production was further confirmed by the nuclear levels of p-Smad2/3 and Smad4. Presence of santamarine (10 µM) reverted the UVA-induced decrease in nuclear p-Smad2/3 and Smad4 levels (Figure 7b).

### 3.9. Santamarine Ameliorated UVA-Induced Deterioration of Nrf-Dependent Antioxidant Mechanism

**Figure 8:**
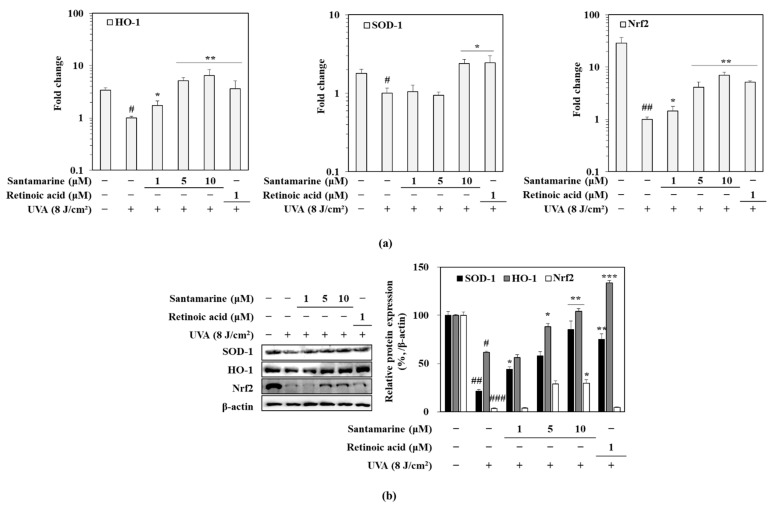
Effect of santamarine on Nrf2-dependent intracellular antioxidant mechanism. The mRNA expression (**a**) and total protein levels (**b**) of Nrf2 and dependent antioxidant enzymes SOD-1 and HO-1 were analyzed using RT-qPCR and Western blot, respectively, after 24 h treatment in UVA-irradiated (8 J/cm^2^) HDFs. β-actin was used as a loading control. Values are means ± SD (*n* = 3). * *p* < 0.05, ** *p* < 0.01 and *** *p* < 0.001 vs. UVA-irradiated untreated control. ^#^ *p* < 0.05, ^##^ *p* < 0.01 and ^###^ *p* < 0.001 vs. non-irradiated control. Retinoic acid was used as a positive control.

To evaluate the antioxidant mechanism of santamarine against UVA-mediated changes in HDFs, mRNA and protein levels of antioxidant enzymes HO-1 and SOD-1 were investigated, along with their upstream activator Nrf2. Results showed that 24 h after UVA exposure, the mRNA expression levels of Nrf2, SOD-1 and HO-1 were significantly diminished (Figure 8a). Similar trends were also observed at protein levels (Figure 8b). The 10 µM santamarine treatment dramatically increased the both mRNA and protein levels of SOD-1 and HO-1 compared to UVA irradiated only group. In addition, levels of SOD-1 and HO-1 were higher than that of non-irradiated untreated control group (Figure 8a). Moreover, santamarine relieved the UVA-induced suppression on Nrf2 mRNA expression. The effect of santamarine on protein levels of Nrf2 and antioxidant SOD-1 and HO-1 enzymes were also similar (Figure 8b).

## 4. Discussion

Natural antioxidants, which have been shown to have multiple benefits on human health apart from their antioxidative properties, are among the first choices of therapeutic agents against UV-induced skin damages. UVA-induced elevation of ROS in skin cells leads to several harmful changes in the intracellular mechanisms including but not limited to overactivation of signaling pathways such as MAPK, DNA damage and dysregulated enzymatic activities [22]. The current study was carried out to evaluate the anti-photoaging properties of reynosin and santamarine, two known sesquiterpene lactones isolated from *A. scoparia*, in UVA-irradiated HDFs.

Photoaging is the premature aging of the skin due to chronic exposure to UVA and accumulation of UVA-induced damages. Negative alterations in the skin connective tissue, mainly due to UVA-induced expressions of MMPs which in turn cleaves the ECM components, is the main culprit for the characteristics of photoaging: loss of skin structure and formation of wrinkles. The current results showed that UVA-irradiation led to upregulated expression of MMP-1 and MMP-9. This was also reported in previous studies and credited to be an outcome of UVA-induced ROS generation [23,24]. Retinoic acid is a known chemical with skin protective properties with antioxidant activity. Therefore, it was used as a positive control in the current study. Although both reynosin and santamarine was shown to possess antioxidant properties, reynosin failed to exert a promising MMP-1 suppression activity to grant further focus for the anti-photoaging evaluation.

On the other hand, santamarine significantly reverted the UVA-induced stimulation of MMP-1 and MMP-9 expressions. It has been long known that the activation of MAPK/AP-1 pathway is the main regulator of the UV-mediated overexpression of MMPs [25]. Therefore, the effect of santamarine on the MAPK/AP-1 signaling was evaluated through activation of p-38, ERK and JNK MAPKs and their downstream targets c-Fos and c-Jun which together form the AP-1 transcription factor. Choi et al. [26] reported that santamarine exhibited anti-inflammatory properties via inhibition of Nf-kB and TNFα-related pathways. Both pathways have been suggested to be partly regulated by AP-1 transcriptional activities as well [27,28]. In the present study, santamarine suppressed the UVA-induced MAPK and AP-1 activation in HDFs shown as suppressed p38 and JNK phosphorylation and decreased nuclear translocation of p-c-Fos and p-c-Jun. It was suggested that the suppression of MAPK-mediated AP-1 activation led to the downregulation of MMP-1 by santamarine. However, santamarine treatment did not suppress the ERK phosphorylation in contrast to studies with other sesquiterpenes with anti-inflammatory and MAPK suppressing activities [26,29]. Moreover, santamarine upregulated the ERK phosphorylation. Cheng et al. [30] and Lin et al. [31] suggested that ERK activation had roles in TGF-β-mediated collagen production. Therefore, the possible collagen production stimulatory effect of santamarine was assessed through TGF-B/Smad pathway analysis and fluorescence staining of intracellular collagen.

One of the characteristics of photoaging is diminished collagen production along with increased collagen degradation due to overexpression of MMPs. The current results confirmed that HDFs produced dramatically low type I procollagen following UVA exposure. Presence of santamarine following UVA irradiation relieved the suppression on collagen production and reverted it close to normal levels compared to non-irradiated cells. Considering that santamarine stimulated ERK phosphorylation and type I procollagen production, its effect of TGF-β and its downstream activators Smad2/3 complex was evaluated. TGF-β signaling regulates the production of type I procollagen via phosphorylation of Smad2/3 complex which in turn form another complex with Smad4 and translocate into nucleus to initiate collagen expression [32]. The current results confirmed that UVA-irradiation of HDFs resulted in significantly low TGF-β levels and suppressed activation of Smad2/3 complex. On the other hand, UVA irradiation increased the levels of Smad7 which is a natural negative regulator of TGF-β/Smad signaling [33]. Adding santamarine to cell culture after UVA irradiation, reverted the inhibitory effects of UVA on TGF-β signaling. Nuclear levels of p-Smad2/3 and Smad4 levels were notably higher after santamarine treatment compared to UVA irradiation only group. Overall, the results suggested that santamarine inhibited the UVA-induced MMP expression via suppression of MAPK/AP-1 pathway while relieving the inhibition of type I procollagen production through activation ERK-mediated TGF-β/Smad signaling.

UVA-stimulated ROS generation also leads to progressive impairment of cellular mechanisms via accumulation and chronic exposure. The cellular antioxidant mechanisms against ROS were shown to be upregulated following UVA irradiation. Rysava et al. [12] showed that skin cells exhibit increasing Nrf2 activation levels, which is the main regulator of cellular antioxidant mechanisms against UV irradiation, peaking around 6 h following UVA exposure. However, after the peak, Nrf2 levels were shown to gradually decrease to significantly lower levels than that of non-irradiated cells. It was confirmed by current results that UVA-irradiated HDFs exerted significantly low levels of Nrf2 and its downstream products, SOD-1 and HO-1, after 24 h of UVA exposure. Choi et al. [26] suggested that santamarine showed its anti-inflammatory properties in LPS-stimulated macrophages via inducing HO-1 expression, which is regulated by Nrf2. The current results were in agreement, as the presence of santamarine reverted the UVA-induced suppression on Nrf2-mediated antioxidant response, shown as stimulated levels of Nrf2, SOD-1 and HO-1 expression both at the mRNA and protein levels.

## 5. Conclusions

In conclusion, the results of the current study suggested that santamarine relieved HDFs from UVA-induced photoaging-related changes in MMP expression and type I procollagen production. Santamarine exerted its effects against UVA via suppression of MAPK/AP-1 and stimulation of TGF-B/Smad signaling pathways. In addition, santamarine exerted antioxidant activities in the UVA-irradiated HDFs. Overall, the present study provides critical evidence for the future application of santamarine as an anti-photoaging agent acting against UVA-induced oxidative stress and related complications. 

## Figures and Tables

**Table 1:** ^1^H and ^13^C NMR spectral data for reynosin *.

Position	δ_H_	δ_C_
1	3.49 (1H, dd, *J* = 11.6, 4.5 Hz)	78.8
2	1.80, 1.56 (1H, m)	32.1
3	2.32 (1H, ddd, *J* = 13.5, 4.9, 1.8 Hz), 2.15 (1H, td, *J* = 13.5, 5.1) 2.15 (1H, td, *J* = 13.5, 5.1)	34.8
4		145.1
5	2.24 (1H, br d, *J* = 10.9 Hz)	54.1
6	4.10 (1H, t, *J* = 10.9 Hz)	81.5
7	2.61 (1H, tdd, *J* = 11.2, 3.1, 3.0 Hz) 5	50.7
8	2.08, 1.60 (1H, m)	22.4
9	2.05 (1H, m), 1.38 (1H, td, *J* = 13.3, 3.8)	36.9
10		44.2
11		141.3
12		171.7
13	6.00 (1H, d, *J* = 3.1 Hz), 5.49 (1H, d, *J* = 3.0 Hz)	117.3
14	0.80 (3H, s)	12
15	4.95, 4.79 (1H, s)	110.2

* Measured in CD_3_OD at 600 and 151 MHz, respectively. Assignments were aided by ^1^H COSY, gHSQC and gHMBC experiments.

**Table 2:** ^1^H and ^13^C NMR Spectral Sdata for Santamarine *.

Position	δ_H_	δ_C_
1	3.49 (1H, dd, *J* = 11.6, 4.5 Hz)	78.8
2	1.80, 1.56 (1H, m)	32.1
3	2.32 (1H, ddd, *J* = 13.5, 4.9, 1.8 Hz), 2.15 (1H, td, *J* = 13.5, 5.1) 2.15 (1H, td, *J* = 13.5, 5.1)	34.8
4		145.1
5	2.24 (1H, br d, *J* = 10.9 Hz)	54.1
6	4.10 (1H, t, *J* = 10.9 Hz)	81.5
7	2.61 (1H, tdd, *J* = 11.2, 3.1, 3.0 Hz) 5	50.7
8	2.08, 1.60 (1H, m)	22.4
9	2.05 (1H, m), 1.38 (1H, td, *J* = 13.3, 3.8)	36.9
10		44.2
11		141.3
12		171.7
13	6.00 (1H, d, *J* = 3.1 Hz), 5.49 (1H, d, *J* = 3.0 Hz)	117.3
14	0.80 (3H, s)	12
15	4.95, 4.79 (1H, s)	110.2

* Measured in CD_3_OD at 600 and 151 MHz, respectively. Assignments were aided by ^1^H COSY, gHSQC and gHMBC experiments.

## Data Availability

All data used to support the findings of this study are available from the corresponding author upon reasonable request.

## Supplementary Materials

The following are available online, Figure S1: ^1^H-^1^H COSY spectrum of reynosin, Figure S2: gHSQC (left) and gHMBC (right) spectra of reynosin, Figure S3: ^1^H-^1^H COSY spectrum of santamarine, Figure S4: gHSQC (left) and gHMBC (right) spectra of santamarine, Figure S5: (**a**) ^1^H-NMR spectrum of reynosin, (**b**) ^13^C-NMR spectrum of reynosin, Figure S6: (**a**) ^1^H-NMR spectrum of santamarine, (**b**) ^13^C-NMR spectrum of santamarine.

## Sample Availability

Samples of the compounds reynosin and santamarine are available from the corresponding author upon reasonable request.
