# Supplementary material for: Santamarine Shows Anti-Photoaging Properties via Inhibition of MAPK/AP-1 and Stimulation of TGF-β/Smad Signaling in UVA-Irradiated HDFs
Source: Molecules. 2021 Jun 11;26(12):3585. doi: 10.3390/molecules26123585 (PMC8230857; doi:10.3390/molecules26123585)
Supplement: Supplementary file 1 [file molecules-26-03585-s001.zip › molecules-1243577-supplementary.pdf]

Santamarine Shows Anti-Photoaging Properties via Inhibition of MAPK/AP-1 and Stimulation of TGF-β/Smad Signaling in UVA-Irradiated HDFs

Authors: Jung Hwan Oh, Junse Kim, Fatih Karadeniz, Hye Ran Kim, So Young Park, Youngwan Seo and Chang-Suk Kong

Supplementary Material – Spectrum analyses for the compounds reynosin and santamarine

Figure S1. 1H-1H COSY spectrum of reynosin.
Figure S2. gHSQC (left) and gHMBC (right) spectra of reynosin.
Figure S3. 1H-1H COSY spectrum of santamarine.
Figure S4. gHSQC (left) and gHMBC (right) spectra of santamarine.
Figure S5. 1H-NMR (a) and 13C-NMR (b) spectra of reynosin.
Figure S6. 1H-NMR (a) and 13C-NMR (b) spectra of santamarine.

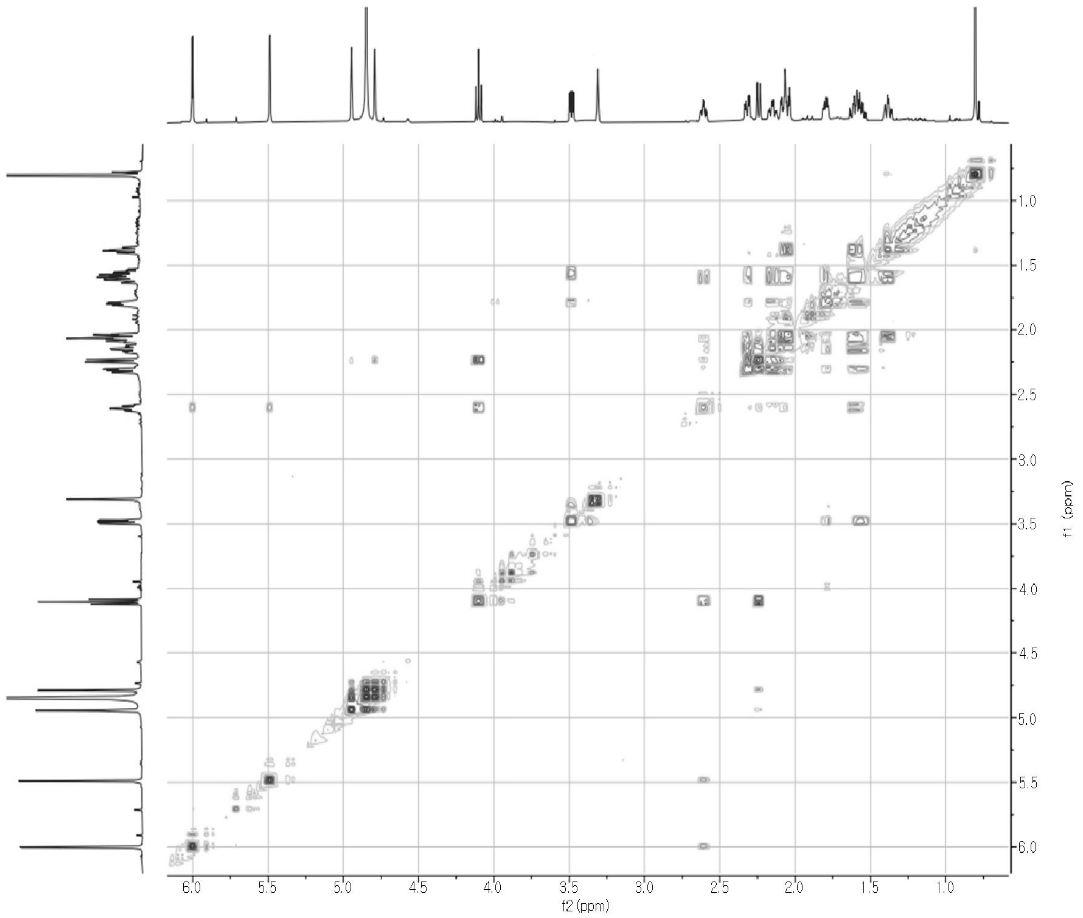

Figure S1. 1H-1H COSY spectrum of reynosin.

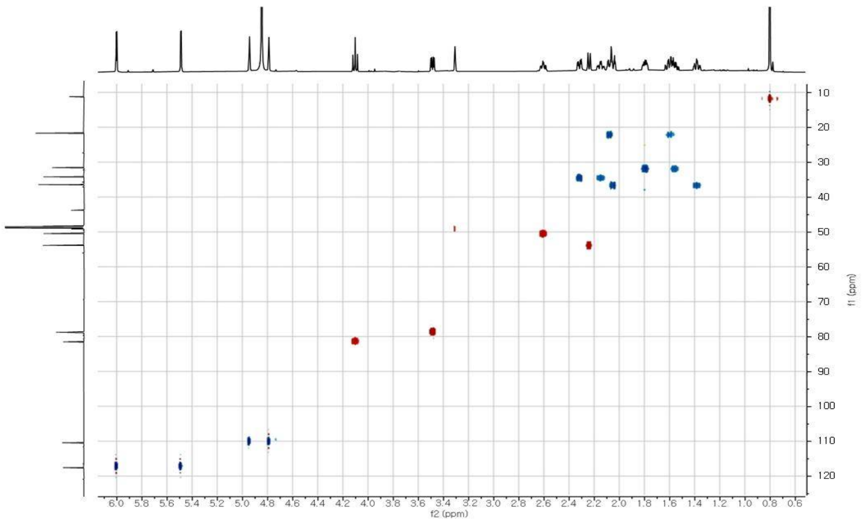

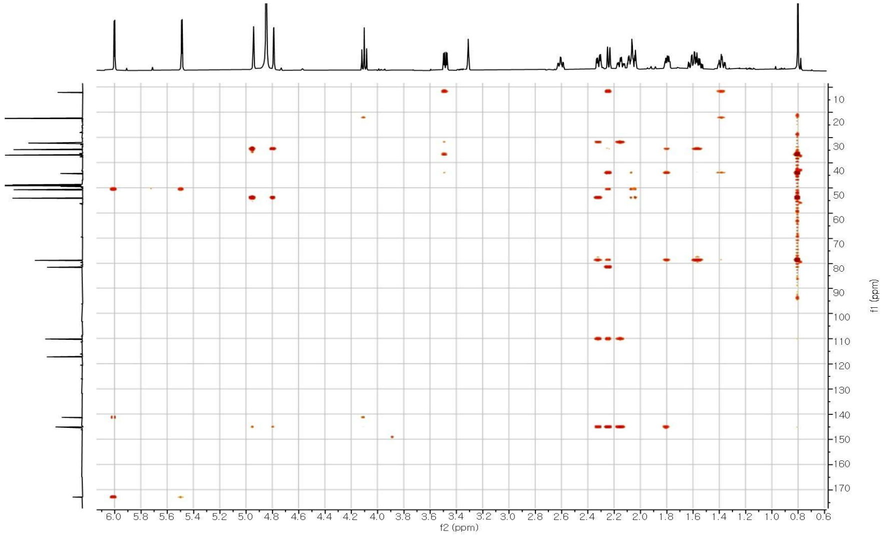

Figure S2. gHSQC (left) and gHMBC (right) spectra of reynosin

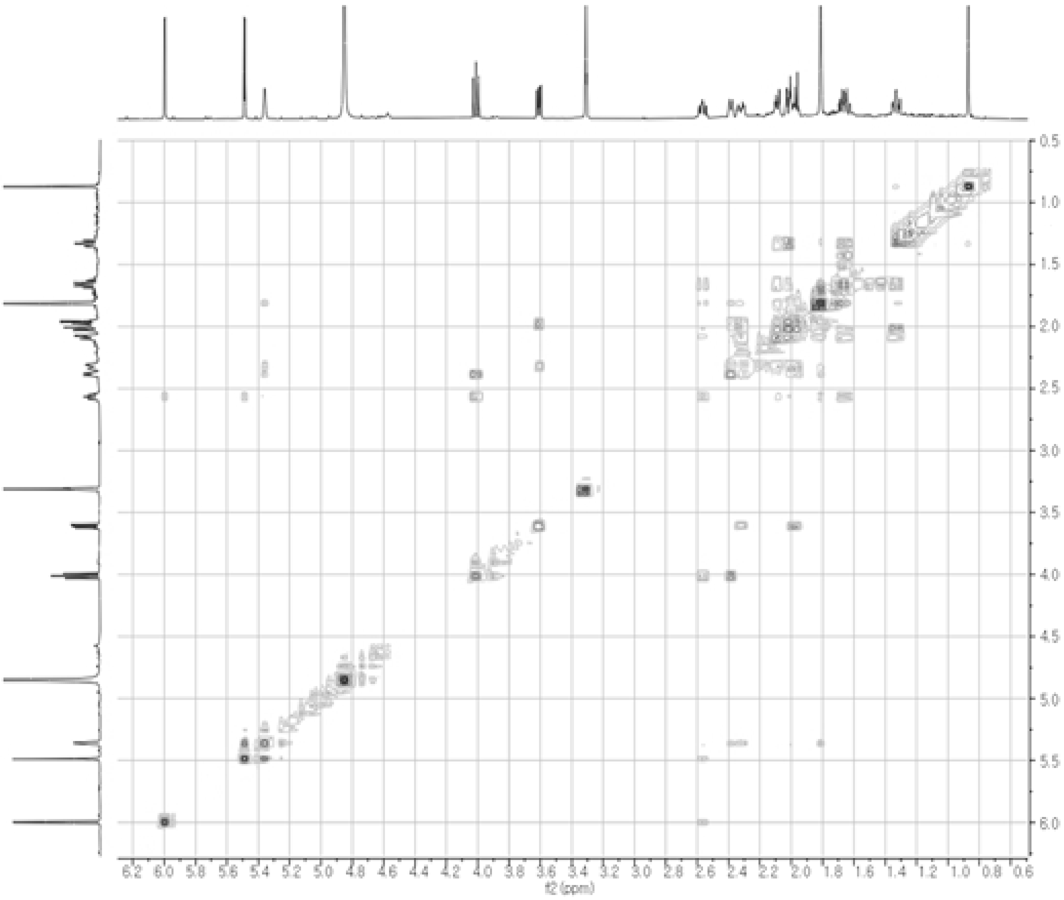

Figure S3. 1H-1H COSY spectrum of santamarine

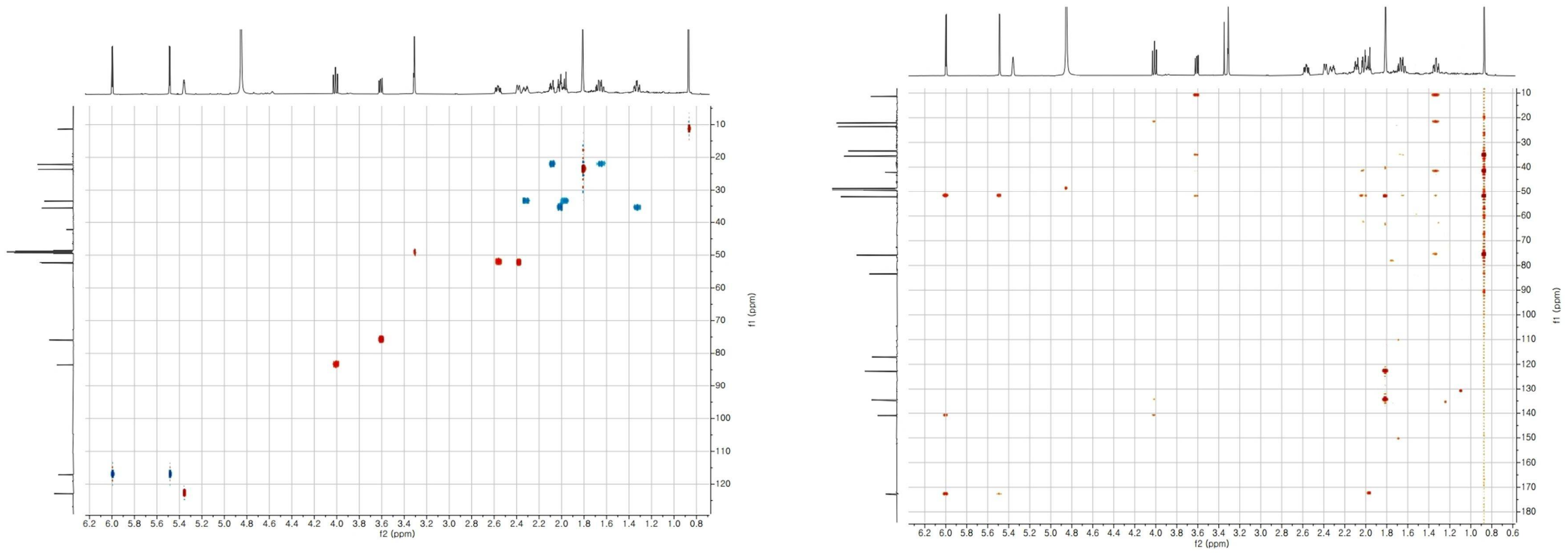

Figure S4. gHSQC (left) and gHMBC (right) spectra of santamarine

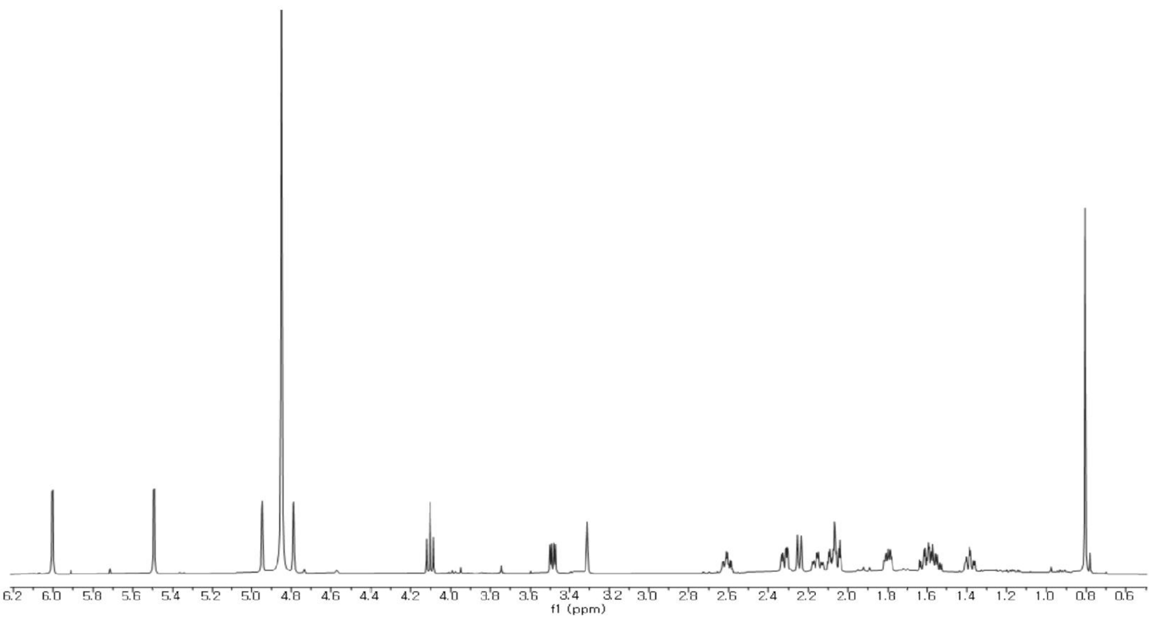

Figure S5a. 1H-NMR spectrum of reynosin.

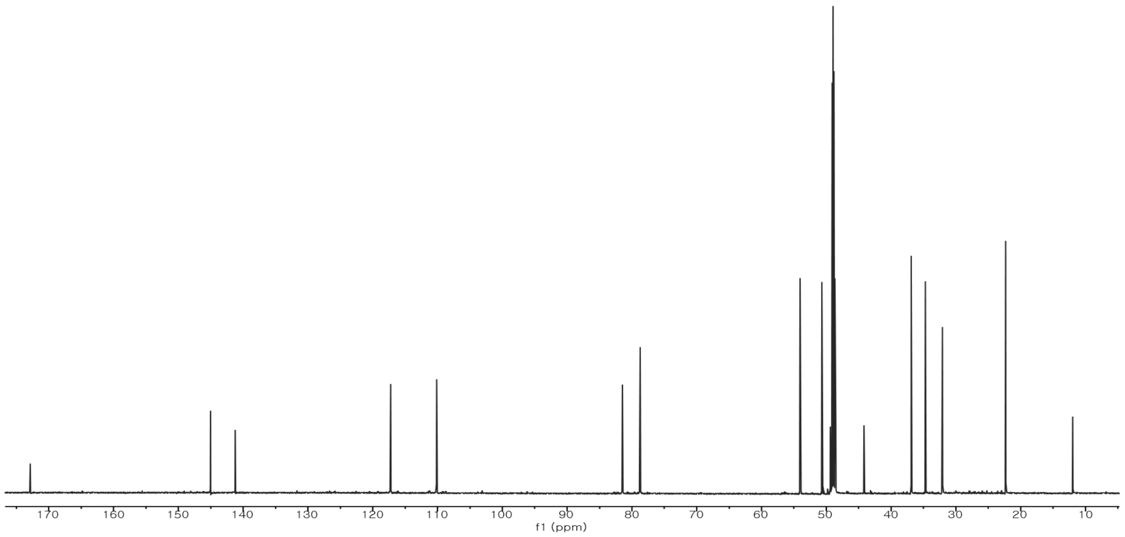

Figure S5b. 13C-NMR spectrum of reynosin

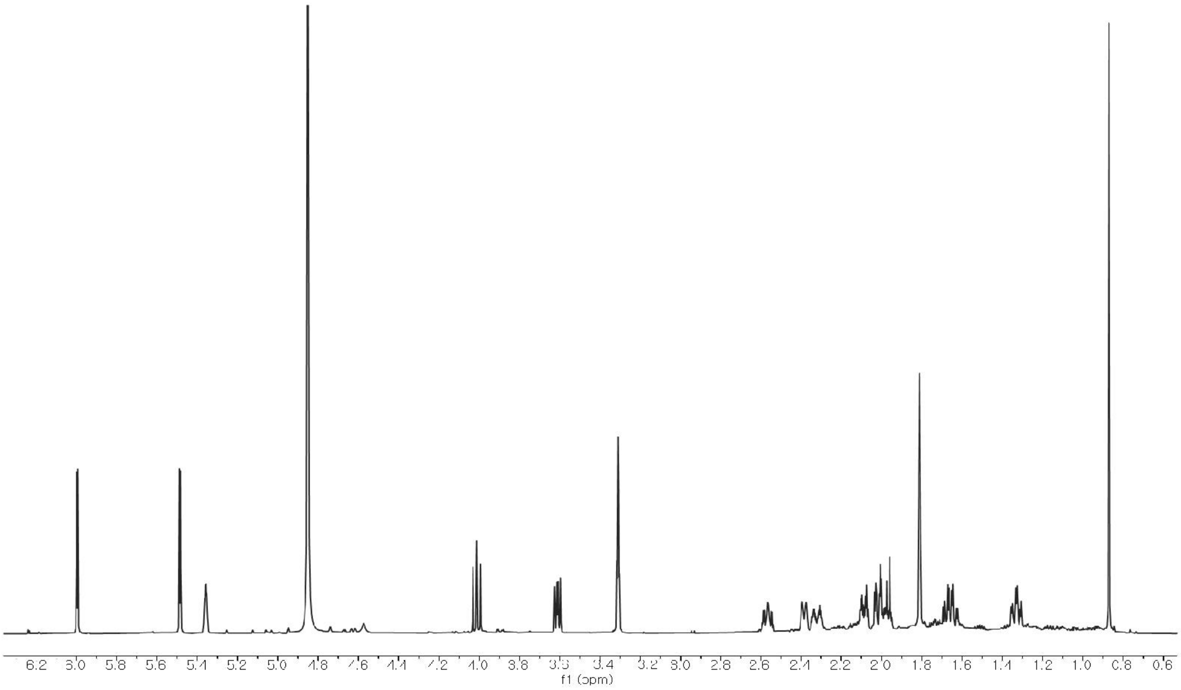

Figure S6a. 1H-NMR spectrum of santamarine

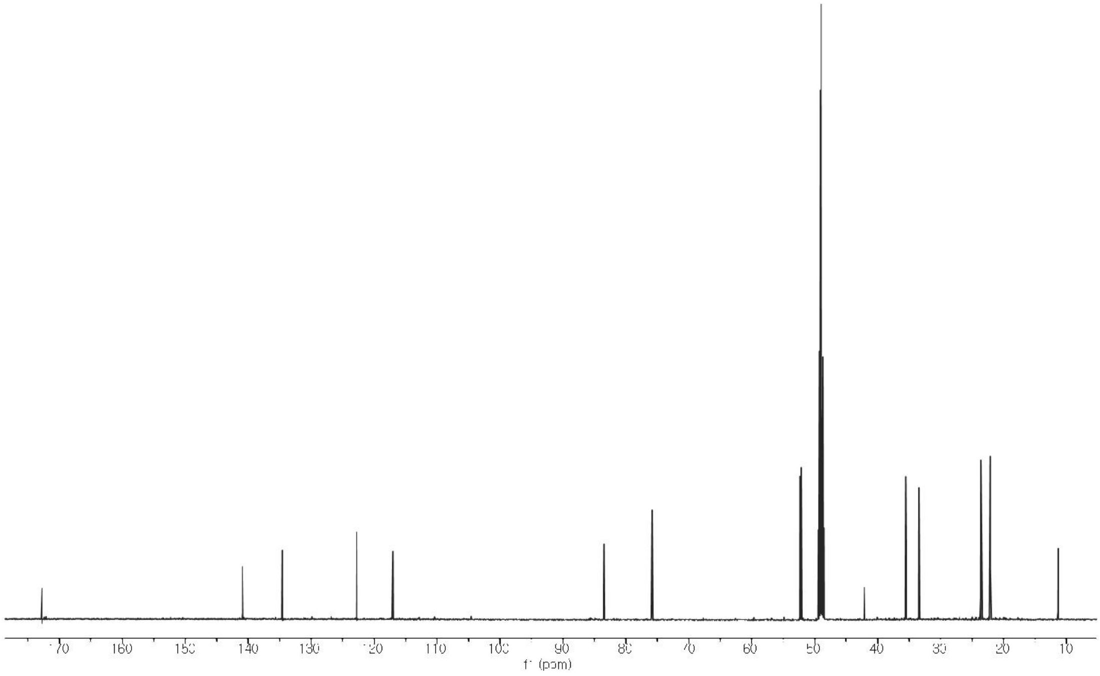

Figure S6b. 13C-NMR spectrum of santamarine
